# Neural stem cells derived from primitive mesenchymal stem cells reversed disease symptoms and promoted neurogenesis in an experimental autoimmune encephalomyelitis mouse model of multiple sclerosis

**DOI:** 10.1186/s13287-021-02563-8

**Published:** 2021-09-09

**Authors:** Christina Brown, Christina McKee, Sophia Halassy, Suleiman Kojan, Doug L. Feinstein, G. Rasul Chaudhry

**Affiliations:** 1 Department of Biological Sciences, Oakland University, Rochester, MI 48309 USA; 2 OU-WB Institute for Stem Cell and Regenerative Medicine, Rochester, MI 48309 USA; 3 Ascension Providence Hospital, Southfield, MI 48075 USA; 4 Department of Neuroscience, OUWB School of Medicine, Oakland University, Rochester, MI 48309 USA; 5 Department of Anesthesiology, The University of Illinois at Chicago, Chicago, IL 60607 USA; 6 Department of Veterans Affairs, Jesse Brown VA Medical Center, Chicago, IL 60612 USA

**Keywords:** Multiple sclerosis, Neural stem cells, Mesenchymal stem cells, Experimental autoimmune encephalomyelitis, Anti-inflammation, Remyelination, Neuroprotection, Neurogenesis

## Abstract

**Background:**

Multiple sclerosis (MS) is an autoimmune inflammatory disease of the central nervous system (CNS). MS affects millions of people and causes a great economic and societal burden. There is no cure for MS. We used a novel approach to investigate the therapeutic potential of neural stem cells (NSCs) derived from human primitive mesenchymal stem cells (MSCs) in an experimental autoimmune encephalomyelitis (EAE) mouse model of MS.

**Methods:**

MSCs were differentiated into NSCs, labeled with PKH26, and injected into the tail vein of EAE mice. Neurobehavioral changes in the mice assessed the effect of transplanted cells on the disease process. The animals were sacrificed two weeks following cell transplantation to collect blood, lymphatic, and CNS tissues for analysis. Transplanted cells were tracked in various tissues by flow cytometry. Immune infiltrates were determined and characterized by H&E and immunohistochemical staining, respectively. Levels of immune regulatory cells, Treg and Th17, were analyzed by flow cytometry. Myelination was determined by Luxol fast blue staining and immunostaining. In vivo fate of transplanted cells and expression of inflammation, astrogliosis, myelination, neural, neuroprotection, and neurogenesis markers were investigated by using immunohistochemical and qRT-PCR analysis.

**Results:**

MSC-derived NSCs expressed specific neural markers, NESTIN, TUJ1, VIMENTIN, and PAX6. NSCs improved EAE symptoms more than MSCs when transplanted in EAE mice. Post-transplantation analyses also showed homing of MSCs and NSCs into the CNS with concomitant induction of an anti-inflammatory response, resulting in reducing immune infiltrates. NSCs also modulated Treg and Th17 cell levels in EAE mice comparable to healthy controls. Luxol fast blue staining showed significant improvement in myelination in treated mice. Further analysis showed that NSCs upregulated genes involved in myelination and neuroprotection but downregulated inflammatory and astrogliosis genes more significantly than MSCs. Importantly, NSCs differentiated into neural derivatives and promoted neurogenesis, possibly by modulating BDNF and FGF signaling pathways.

**Conclusions:**

NSC transplantation reversed the disease process by inducing an anti-inflammatory response and promoting myelination, neuroprotection, and neurogenesis in EAE disease animals. These promising results provide a basis for clinical studies to treat MS using NSCs derived from primitive MSCs.

**Supplementary Information:**

The online version contains supplementary material available at 10.1186/s13287-021-02563-8.

## Background

Multiple sclerosis (MS) is a chronic autoimmune inflammatory disease of the central nervous system (CNS) [1]. MS affects approximately 400,000 individuals in the USA and 2.5 million people worldwide. As a result, it inflicts a substantial economic and societal burden due to the early age of disease onset and recurrent relapses [2]. In MS, inflammatory responses are elicited by the intrinsic immune system and parenchymal glial cells, which contribute to oligodendrocyte damage, demyelination, axonal damage, and impaired or reduced neuronal signaling [1]. Multiple factors, including genetics and environmental agents, seem to be involved with the onset of MS [1]. Although drug treatments help reduce disease progression or minimize disability in patients, they cause severe side effects and do not reverse the symptoms of MS [3]. Therefore, it is imperative to design therapeutic strategies that stop disease progression and repair the damage to the CNS without severe side effects to MS patients. With the advent of recent progress in stem cell research, cell therapy is considered a more promising alternative to drug therapy [4]. Mesenchymal stem cells (MSCs) have shown paracrine effects by secreting immunomodulatory cytokines and trophic factors [5]. MSCs are known to home towards the injury site and participate in the healing and repair processes throughout life [6]. Therefore, there is tremendous interest in MSCs due to their medicinal properties. However, MSCs isolated from adult sources have poor growth as well as limited self-renewal and differentiation potential and pose a significant risk of graft verse host disease (GvHD) [6]. On the other hand, MSCs isolated from perinatal sources are more naïve, exhibiting a low risk of host immune response due to the lack of expression of the major histocompatibility complex (MHC) class II antigens, and thus do not cause GvHD [6]. Recently, we have isolated highly proliferative primitive MSCs, which can be rapidly amplified to large numbers, and differentiated into functional derivatives, for large-scale animal and clinical studies [6–8]. MSCs are known to have homing capabilities to the site of injury as well as the ability to secrete cytokines and growth factors that may indirectly promote endogenous tissue regeneration [5]. However, the ability of transplanted MSCs to cross the blood–brain barrier (BBB) to repair the damaged CNS is not well established [9]. Several reports describe the ability of neural stem cells (NSCs) to migrate to the area of pathology in experimental models of CNS diseases [10, 11]. In addition, resident NSCs that produce new neurons throughout life are continuously decreased in MS patients [12]. Therefore, stem cell-based therapies that promote endogenous neural regeneration to repair the damage in the CNS [13, 14] may prove ideal for the treatment of MS. In this study, we applied a novel cell therapy approach whereby highly proliferative and naïve primitive MSCs were first differentiated into NSCs that were transplanted into an experimental autoimmune encephalitis (EAE) mouse model, which is known to share similar characteristics and pathological features with MS [15]. Our results demonstrated that EAE disease symptoms were reduced or reversed and significant improvements were observed in the pathology of the CNS in animals transplanted with MSCs and NSCs, with the latter being the more effective. The transplanted cells survived and migrated to the CNS where they promoted myelination and neural protection by modulating immune response and inhibiting astrogliosis as well as induced endogenous neurogenesis, thereby leading to functional recovery. Despite being highly proliferative, neither MSCs nor their derivatives, NSCs, caused immune rejection or teratoma formation. These studies demonstrate that NSCs derived from primitive MSCs have therapeutic potential to treat MS and other neurodegenerative diseases.

## Materials and methods

### Maintenance and culture of primitive MSCs

Previously isolated and characterized naïve primitive MSCs [8] were maintained using growth medium (GM) containing DMEM nutrient mix F12 medium (DMEM/F12; Life Technologies, Carlsbad, CA, USA), supplemented with 10% fetal bovine serum (FBS; VWR, Radnor, PA, USA), and 5.6% of antibiotic solution (0.1% gentamicin, 0.2% streptomycin, and 0.12% penicillin) (Sigma-Aldrich, St Louis, MO, USA) and incubated at 37 °C in an atmosphere of 5% CO_2_ in a humidified incubator. Using this medium, primitive MSCs were maintained and expanded into large numbers without losing their self-renewal and differentiation potential.

### Differentiation of primitive MSCs into NSCs and oligodendrocytes cells (ODCs)

Primitive MSCs were induced towards the neural lineage by culturing the cells in induction media containing 10 µg epidermal growth factor (EGF; PeproTech, Rocky Hill, NJ, USA) in DMEM/F12 basal medium supplemented with 5.6% antibiotic solution for 3 days. Then, it is replaced with neural media containing 20 µg EGF, 20 ng basic fibroblast growth factor (bFGF), 2 mM Glutamine (Sigma-Aldrich), 1 × B27 supplement (Thermo Fisher Scientific, Waltham, MA, USA), in the neurobasal medium for 2 weeks. NSCs were differentiated into ODCs by culturing in neurobasal medium containing 2% B27, 10 ng bFGF, 10 ng PDGF-AA (PeproTech), and 100 ng SHH (PeproTech) for 2 weeks and then 0.5% FBS, 2% B27, 1 × N2, and 30 ng T3 (Thermo Fisher Scientific) for an additional 10 days. Characterization of NSCs and ODCs for the expression of neural and ODC markers was performed by flow cytometry, quantitative reverse transcriptase-polymerase chain reaction (qRT-PCR), immunocytochemical staining, and western blot analyses.

### Flow cytometry analysis

Cells were grown to 70% confluency, trypsinized, washed with PBS, and pelleted. Cells were stained against CD44 and CD90 (FITC labeled antibodies) or CD29, CD73, CD105 (APC labeled antibodies) (Becton Dickinson, Franklin Lakes, NJ, USA) and analyzed using FACS Canto II (Becton Dickinson) and Diva Software (Becton Dickinson). APC- and FITC-labeled mouse IgG were used as negative controls.

### Immunocytochemical analysis

Cells were fixed with 4% paraformaldehyde for 10 min at room temperature, permeabilized with 0.5% Triton X-100 (Sigma-Aldrich), and blocked with 2% bovine serum albumin (Sigma-Aldrich) for 1 h. Cells were then treated with primary antibodies at 1:100 dilution: NESTIN, TUJ1, VIMENTIN, PAX6, OLIG2, SOX10, O4, MBP, and MOG at 4 °C overnight, followed by staining with secondary antibodies at 1:200 dilution at room temperature for 2 h. Cells were counterstained with DAPI at 1:100 dilution for 5 min at room temperature. Fluorescent images were captured using a confocal microscope (NIKON Instruments Inc., Melville, NY, USA). The fluorescent intensity and percentage of positive cells were calculated using ImageJ software (NIH, Bethesda, MD, USA). To quantify the fluorescent intensity, the following equation was used, fluorescent intensity = integrated density − (area of selected cell x mean fluorescence of background readings).

### Western blot analysis of cells

Cells were lysed using RIPA buffer (Sigma-Aldrich), and protein was quantified using the Pierce™ 660 nm protein assay kit and NanoDrop 1000 spectrophotometer (Fisher Scientific). The cell lysate (30 μg of total proteins) was resolved using SDS-PAGE with 12% resolving gel and 6% stacking gel and transferred to a nitrocellulose membrane (Bio-Rad, Hercules, CA, USA) at a continuous current of 100 V for 90 min. For antibody staining, the membrane was blocked with 5% nonfat dry milk dissolved in TBS 1X containing 0.1% Tween-20 (TBST) for 30 min and incubated with primary antibodies at a 1:500 dilution in the blocking solution overnight at 4 °C. The membrane was then washed with TBST and incubated with the secondary antibody conjugated with HRP at a 1:10,000 dilution in blocking solution for 2 h at room temperature. After washing with TBST, the blot was stained with Bio-Rad chemiluminescence for 5 min, and bands were visualized using a chemidoc (Bio-Rad). Band intensities were quantified using ImageJ software (NIH, Bethesda, MD, USA) and normalized fold protein expression to GAPDH.

### Induction of EAE in mice

All animal experiments were approved by the Institutional Animal Care and Use Committee (IACUC), Oakland University, Rochester, Michigan (IACUC #18081), and the Institutional Biosafety Committee (IBC), Oakland University, Rochester, Michigan (IBC #2858). 6-week-old C57BL/6J mice were obtained from Jackson Laboratory (Bar Harbor, ME, USA), and EAE was induced by subcutaneous immunization with 200 μg of myelin oligodendrocyte glycoprotein (MOG)_33–35_ peptide in complete Freud’s adjuvant containing 2–5 mg killed mycobacterium tuberculosis H37Ra/mL emulsion (Hooke Laboratories, Lawrence, MA, USA). Mice were also injected intraperitoneally (i.p.) with pertussis toxin (100 ng, Hooke Laboratories) at the day of immunization and 24 h later following the published protocol [16]. Clinical score was blindly registered according to the following scale: 0: no clinical signs, 0.5: partial limp tail, 1: limp tail, 1.5: mild impaired righting, 2: severe impaired righting, 2.5: mild paresis of one hind limb, 3: severe paresis of one hind limb, 3.5: severe paresis of one hind limb and mild paresis of second hind limb, 4: paresis of two hind limbs, 4.5: paresis of both hind limb and partial fore limb, and 5: severe paralysis or death [16]. Non-injected C57BL/6J mice were used as controls. All mice were monitored and assessed for clinical score twice a day and maintained at the Oakland University Animal Facility under a 12-/12-h light and dark cycle.

### Cell transplantation in EAE mice

Cells were labeled with cell membrane labeling dye PKH26 (Sigma-Aldrich) following the manufacturer’s instructions [17]. Confocal microscopy and flow cytometry analyses were used to confirm the efficiency of fluorescently labeling cells prior to transplantation. There were eight treatments (*n* = 6 each), 1. Untreated healthy control, 2, healthy control + MSCs, 3. healthy control + NSCs, 4. Untreated EAE, 5. EAE score 1 + MSCs, 6. EAE score 1 + NSCs, 7. EAE score 2 + MSCs, and 8. EAE score 2 + NSCs. The experiments were performed in triplicates. The mice were restrained in a rodent restrainer for cell transplantation. 10^6^ primitive MSCs or NSCs were injected intravenously via the tail vein at either clinical score 1 or 2. The mice were monitored for 15 days following cell transplantation and then euthanized humanly to collect CNS, lymphoid tissues (spleen, brain, and spinal cord), and blood for analysis.

### Tracking of transplanted cells

Blood and tissue samples were placed in red blood cell lysis buffer for 5 min and then homogenized, except blood samples, in a centrifuge tube. Cell culture medium was then added to the tube, inverted, and centrifuged at 1300 rpm for 5 min at 4 °C. The supernatant was discarded, and cells were resuspended in PBS 1x. The cells were then filtered through a cell strainer. Labeled cells in each sample were determined using the FACS Canto II (Becton Dickinson) and Diva Software (Becton Dickinson).

### Histochemical and immunofluorescence staining

For paraffin embedding, brain and spinal cord tissue samples were fixed with 4% paraformaldehyde for 24 h. They were then dehydrated in 70% ethanol for 1 h, 95% ethanol for 1 h, 100% ethanol for 4 h, xylene for 2 h, and paraffin for 3 days. Tissue samples were then embedded in paraffin and sectioned (5–10 μm thick) using a microtome. The paraffin-embedded samples were stained with H&E (Hematoxylin and eosin; Thermo Fisher Scientific) to evaluate the cellular structure of the tissue and Luxol fast blue (LFB; Sigma-Aldrich) to examine myelination.

To detect the expression of human neural and inflammatory markers expressed in the mouse brain and spinal cord tissue, the sections were stained with primary antibodies 1:100 dilution: CD45, CD68, CD3E, GFAP, MBP, MOG, TUJ1, NESTIN, OLIG2, O4 and HNA (human nuclear antigen), respectively, at 4 °C overnight followed by staining of secondary antibodies at 1:200 dilution at room temperature for 2 h. They were counterstained with DAPI at 1:100 dilution for 5 min at room temperature. Representative fluorescent images/sections of the randomly selected stained sections were captured using a confocal microscope (NIKON Instruments Inc.).

### Quantification of Treg and Th17 cells in the blood, spleen, and CNS tissues

For flow cytometry analysis, the tissue samples were processed as stated above for cell tracking. Isolated cells (10^6^) from each tissue were stained with CD25, IL-17A (FITC- labeled antibodies), CD4 (APC-labeled antibodies), or FOXP3 (PE-labeled antibodies) (Becton Dickinson). Labeled cells were analyzed on a FACS Canto II (Becton Dickinson) using Diva Software (Becton Dickinson).

### T cell proliferation assay

Splenocytes isolated from control and EAE mice were cultured in 96-well plates (2 × 10^5^ cells/well) using RPMI1640 medium (Gibco, Waltham, MA, USA) supplemented with 10% FBS and 5.6% of the antibiotic solution in the presence or absence of MOG peptide (10 µg/ml) at 37 °C in 5% CO_2_ for 72 h. Then, MSCs or NSCs (1 × 10^3^ cells/well) were added and incubated for an additional 24 h. Measurement of T cell proliferation was performed using BrdU proliferation kit (Novus Biologicals, Centennial, CO, USA), according to the manufacturer's instructions.

### qRT-PCR analysis of cells and mouse tissues

Isolation of the total cellular mRNA was performed using the GeneJET RNA purification kit (Thermo Fisher Scientific) following the manufacturer’s instructions. Total RNA was purified with DNase and incubated at 37 °C for 30 min using a thermocycler (Bio-Rad, Hercules, CA, USA). cDNA was synthesized using iScript kit (Bio-Rad), and qRT-PCR was performed by using Sso-Advanced Universal SYBR Green Supermix Kit (Bio-Rad) on CFX96 Real-Time System (Bio-Rad). A 10 µL reaction was used, which included 5 µL Sybr green, 3 µL of distilled water, 0.5 µL of forward primer, 0.5 µL of reverse primer, and 1 µL of 1:10 diluted cDNA. Each reaction was exposed to the following conditions: 98 °C for 10 min, followed by 30 s of 98 °C, 20 s of 60 °C, and 30 s of 72 °C for 44 cycles in 96‐well optical reaction plates (Bio‐Rad). Human and mouse *GAPDH/Gapdh* and *β-ACTIN/β-Actin* genes were used to normalize fold gene expression. Mouse primers were screened using primer BLAST [18] for homology against the human genome and tested against human cells using qRT-PCR. Primer sequences are listed in Additional file 1.

### Experimental design and statistical analysis

Statistical analysis was carried out by using one‐way analysis of variance (ANOVA) test for multiple comparisons followed by post-hoc tests and ANOVA for repeated measures followed by Tukey’s post-hoc analysis. Data are presented as the mean ± standard error of the mean (SEM) of triplicates per analysis. Results were analyzed using SPSS version 26 (SPSS Inc. USA), and *p* values ≤ 0.01 were considered statistically significant. All statistical graphs were created using Microsoft Excel (Microsoft, Redmond, WA, USA).

## Results

### Characterization and differentiation of primitive MSC-derived NSCs

**Fig. 1:**
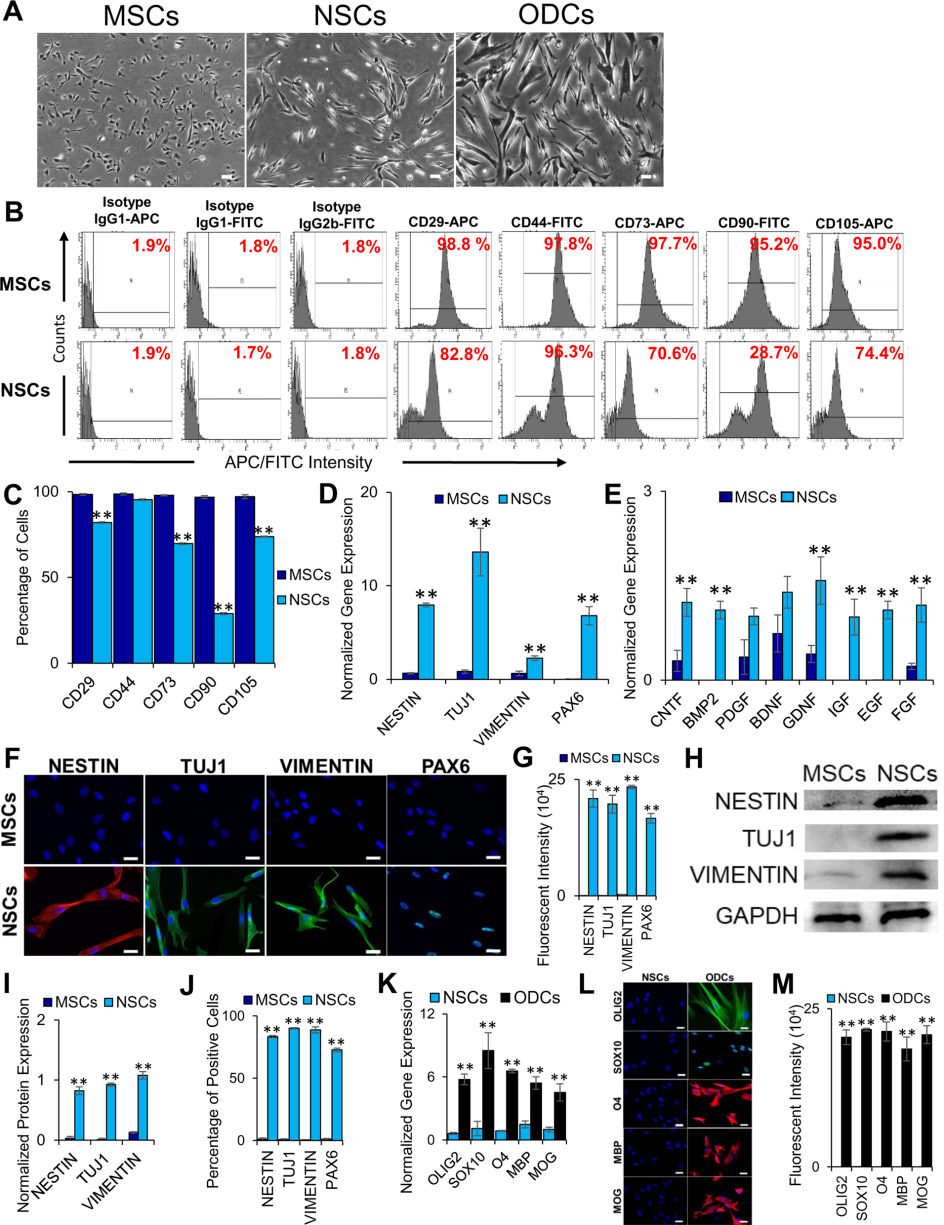
Characterization of NSCs and ODCs derived from primitive MSCs. **a** Phase-contrast images of the primitive MSCs, NSCs, and ODCs. Scale bars represent 100 μm (magnification: 4 ×). **b**, **c** Histograms and graphical representation of the expression of MSC markers as determined by flow cytometry, respectively. Expression of MSC markers, CD29, CD44, CD73, CD90, and CD105, was significantly reduced in NSCs (**p ≤ 0.01). **d**, **e** Expression of neural and neurotrophic genes, respectively, as determined by qRT‐PCR. NSCs expressed neural markers, *NESTIN, TUJ1, VIMENTIN*, and *PAX6*. NSCs also expressed neurotrophic factors, *CNTF, BMP2, PDGF, BDNF, GDNF, IGF, EGF,* and *FGF* at higher levels. **f** Expression of neural proteins as determined by immunocytostaining. Shown are merged images of DAPI (blue) and human antibodies (green and red). NSCs had significantly higher expression of neural proteins, NESTIN, TUJ1, VIMENTIN, and PAX6, than MSCs. **g** Quantification of fluorescent intensity of immunocytostained proteins (**p ≤ 0.01). **h**, **i** Western blot and quantitative analysis of normalized neural protein expression using ImageJ software, respectively (**p ≤ 0.01). All proteins were normalized to GAPDH expression. MSCs expressed low levels of NESTIN and VIMENTIN, but not TUJ1, whereas NSCs had a significantly high level of expression of these markers. **j** Percentage of immunocytostained cells positive for NESTIN, TUJ1, VIMENTIN, and PAX6 (**p ≤ 0.01). **k** Expression of genes, *OLIG2, SOX10, O4, MBP,* and *MOG,* in ODCs as determined by qRT‐PCR. **l** Expression of proteins, OLIG2, SOX10, O4, MBP, and MOG in ODCs, as determined by immunocytostaining. Shown are merged images of DAPI (blue) and human antibodies (green and red). **m** Quantification of fluorescent intensity of immunocytostained proteins (**p ≤ 0.01). Fold gene expression (in **d**, **e**, **k**) was normalized to *GAPDH* and *β-ACTIN,* and error bars represent the SEM of triplicate experiments (**p ≤ 0.01). All scale bars (**f**, **l**) represent 50 μm (magnification: 40 ×)

We first differentiated the primitive MSCs into NSCs [19], which displayed typical neural extension morphology (Fig. 1a), loss of MSC markers (Fig. 1b, c), and expression of the neural genes, *NESTIN*, *TUJ1*, *VIMENTIN*, and *PAX6*, as well as neurotrophic factors, *CNTF*, *BMP2*, *PDGF*, *BDNF*, *GDNF*, *IGF*, *EGF*, and *FGF* (Fig. 1d, e). Similarly, translational expression of several neural proteins, NESTIN, TUJ1, VIMENTIN, and PAX6, was displayed by NSCs (Fig. 1f, g). These results were further validated by western blot analysis showing the expression of the neural proteins in NSCs (Fig. 1h, i). This protocol reproducibly yielded 73% differentiation of MSCs into NSCs (Fig. 1j). The ability of NSCs to differentiate to the glial linage, particularly ODCs (Fig. 1a), was demonstrated by the expression of *OLIG2, SOX10, O4, MBP,* and *MOG* at the transcriptional level (Fig. 1k) and OLIG2, SOX10, O4, MBP, and MOG at the translational level (Fig. 1l, m) by the NSC-derived ODCs. NSCs also differentiated into the neuronal lineage (data not shown).

### Improvement in the neurobehavior and reduction in disease severity in EAE mice transplanted with cells

**Fig. 2:**
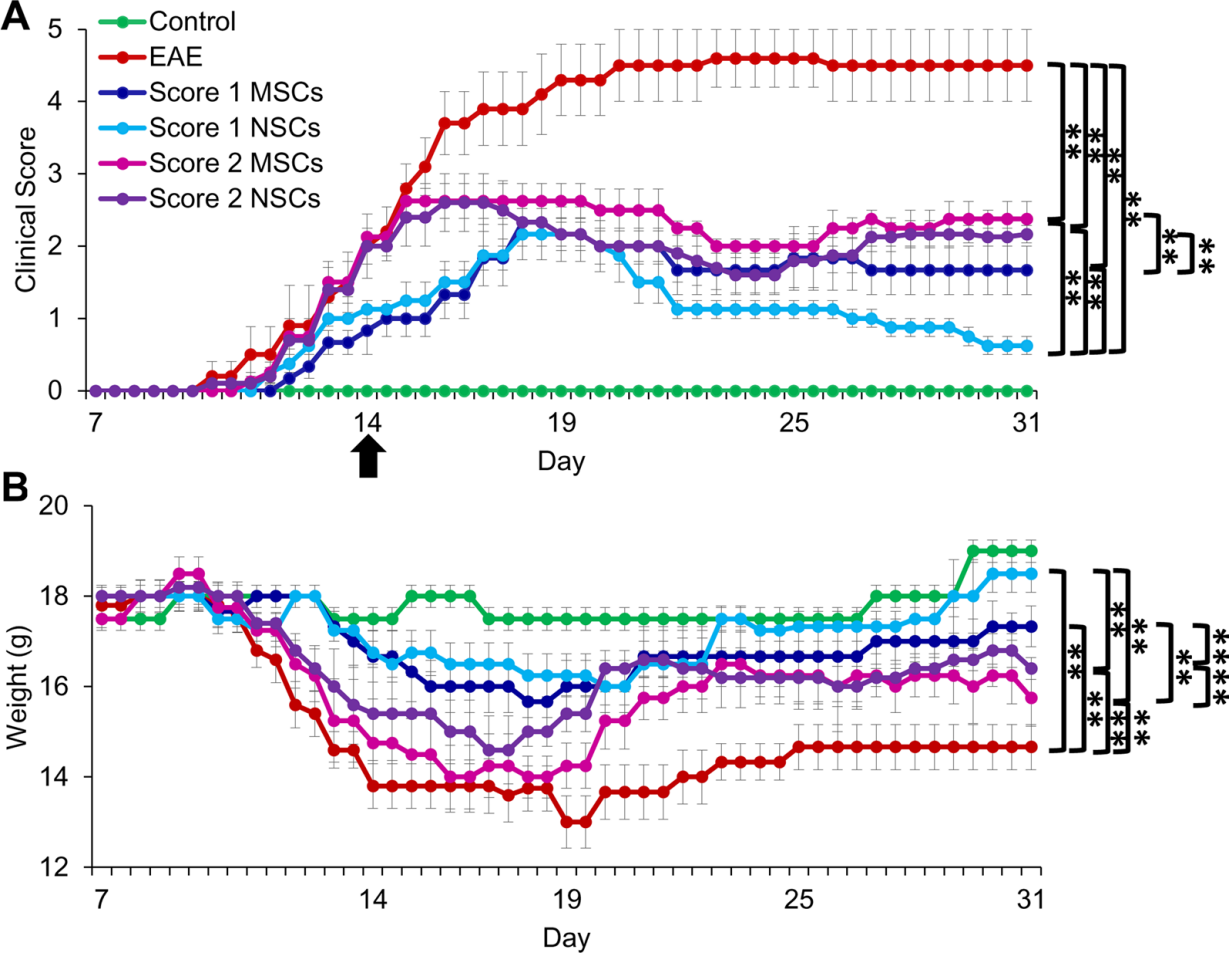
Effect of cell transplantation on the EAE clinical symptoms. **a**, **b** Changes in the clinical score of disease symptoms and weight in EAE mice (averages of *n* = 6 each) following transplantation of cells, respectively. Primitive MSCs and NSCs were transplanted on day 14 (indicated by black arrow) at EAE score 1 and score 2 (**p ≤ 0.01). The animals were monitored, and clinical symptoms as well as weight, were recorded daily

EAE mouse model was used to investigate the effects of MSCs and NSCs on the disease process. EAE was induced in mice by MOG peptide immunization using an established protocol [16]. Disease progression was determined by neurobehavioral analysis according to the well-established disease symptom scale (Additional file 2, Hooke labs). The first signs of EAE induction in animals were noticed 11–12 days after MOG injections. Once the animals reached EAE disease scores 1 and 2, they were intravenously injected with 10^6^ PKH26 labeled cells (Additional file 3a). The animals were evaluated for neurobehavioral changes twice a day until they were sacrificed for post-transplantation analysis. Neurobehavioral analysis results showed that the average clinical score of the EAE controls (4.5 ± 0.5) were significantly higher than the average score of mice transplanted with MSCs (1.7 ± 0.3) and NSCs (0.6 ± 0.1) at score 1 and MSCs (2.4 ± 0.2) and NSCs (2.2 ± 0.1) at score 2 (Fig. 2a and Additional file 3b). In addition, the average weight of the untreated EAE controls (13 g ± 0.6 g) were significantly lower than the average weights of the mice transplanted with healthy controls (Fig. 2b and Additional file 3b), MSCs (15.7 g ± 0. 7 g) and NSCs (16 g ± 0.4 g) at score 1 and MSCs (14 g ± 0.7 g) and NSCs (14.6 g ± 0.7 g) at score 2. When MSCs were transplanted at EAE score 1, they only slowed and halted the disease progression, whereas NSCs slowed and reversed the disease process, and animal condition was improved to near normal levels within two weeks after cell transplantation. Overall, NSCs showed greater efficacy than MSCs when transplanted at both EAE scores 1 and 2, but the results were more significant in the case of score 1. Therefore, we selected EAE score 1 transplanted animals for a detailed post-transplantation investigation. Animals were sacrificed two weeks post-transplantation, and blood, lungs, lymphatic and CNS tissues were collected for histological, biochemical, immunological, and molecular analyses.

### Tracking of transplanted cells

**Fig. 3:**
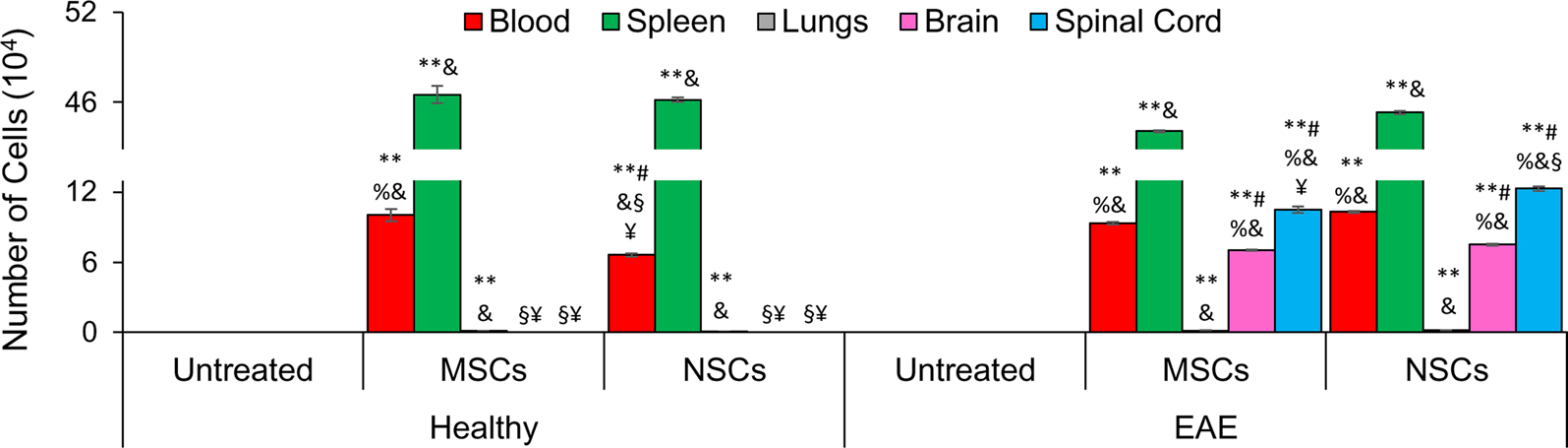
Transplanted cells homed to the blood, spleen, lungs, and CNS. To determine labeled cells in various organs, collected tissue samples prepared as described in Methods and Materials were subjected to flow cytometry. Symbols, **, #, %, &, §, and ¥, indicate significant difference at *p* ≤ 0.01 between all experimental conditions: healthy control, healthy control + MSCs, healthy control + NSCs, EAE, EAE + MSCs, and EAE + NSCs. A significant number of labeled cells was found in the blood and spleen, but not lungs of both healthy and EAE mice. Labeled cells were found in the CNS (both brain and spinal cord) of transplanted EAE animals

The labeled transplanted cells homed to the blood, spleen, brain, and spinal cord (Fig. 3). Only an insignificant number of transplanted cells were found in the lungs of all animals. Interestingly, significantly more transplanted MSCs (10.0%) than NSCs (6.7%) were observed in the blood of healthy animals, suggesting greater survivability of MSCs in the vascular system. In contrast, the number of transplanted MSCs and NSCs was 9.3% and 10.3% in the blood of EAE animals, respectively. Nearly half of the transplanted cells were found in the spleen in all animals. Notably, a significant number of transplanted cells were present in the CNS of EAE animals but not in healthy controls. A similar number of transplanted MSCs and NSCs were found in the brain (7.0% and 7.5%, respectively). Notably, NSCs (12.3%) were significantly more in the spinal cord than MSCs (10.5%). Overall, NSCs exhibited greater survivability in EAE animals than MSCs. Tissue samples were then subjected to histological analysis to investigate the effects of the transplanted cells on immune infiltrates.

### Cell transplantation reduced immune infiltrates in CNS

**Fig. 4:**
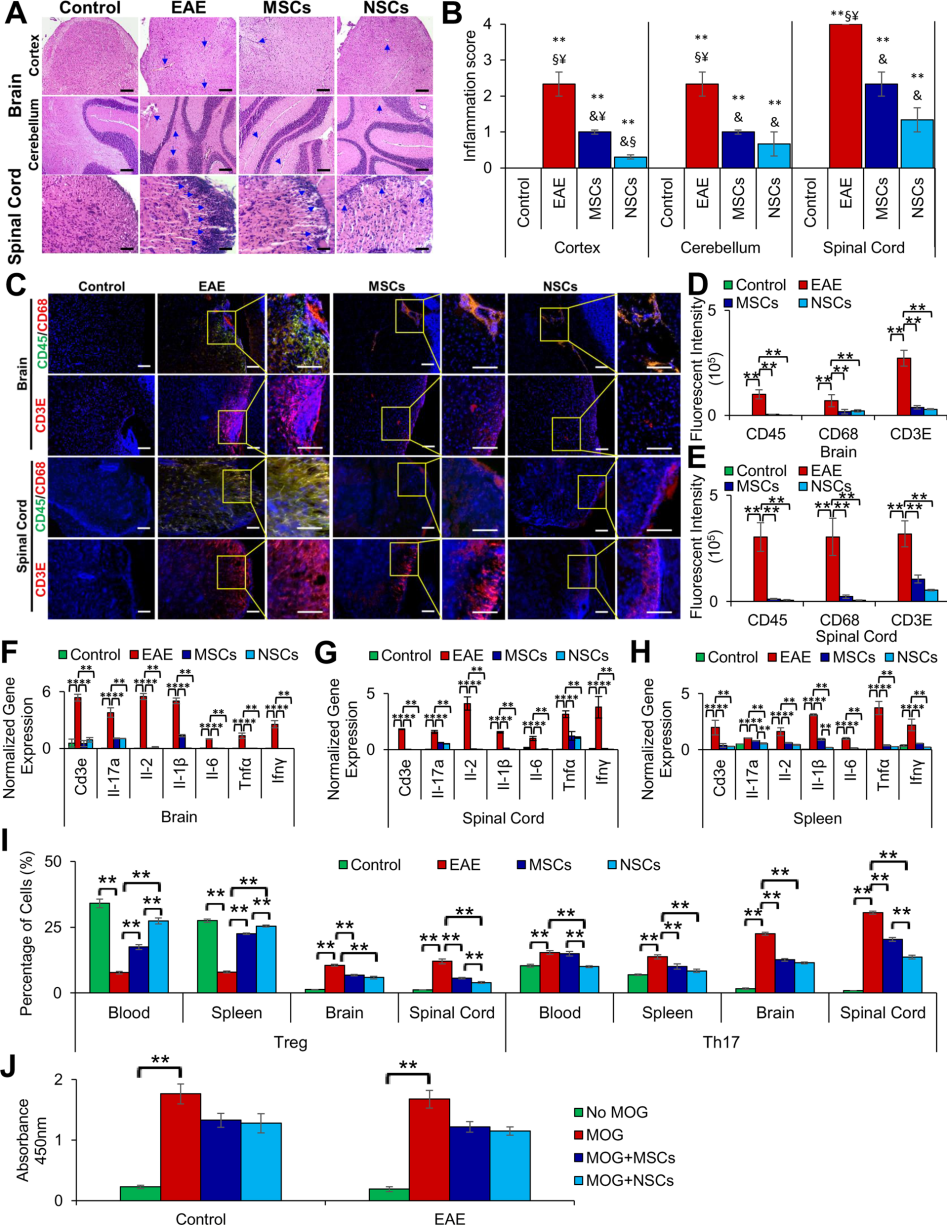
Reduction in inflammation and modulation of Treg and Th17 cells by the transplanted cells. **a**, **b** Histological analysis of paraffin sections stained with H&E and inflammation scores of the brain and spinal cord, respectively. Cellular infiltrates reduced significantly in the CNS in EAE animals transplanted with MSCs and NSCs. Symbols, **, &, §, and ¥ indicate significant difference at *p* ≤ 0.01 between all experimental conditions: healthy control, EAE, EAE + MSCs, and EAE + NSCs. All scale bars represent 50 μm (magnification: 40 ×). **c** Immunohistochemical staining of paraffin sections of the brain and spinal cord with cell-specific antibodies, CD45, CD68, and CD3E, representing leukocytes, macrophages, and T cells, respectively. The inserts (on the right) were magnified and overexposed to enhance contrast. All scale bars represent 50 μm (magnification: 40 ×). **d**, **e** Quantification of fluorescent intensity of brain and spinal cord sections shown in **c**, respectively (**p ≤ 0.01). **f**–**h** Transcription of inflammation markers, *Cd3e, Il-17a, Il-2, Il-1β, Il-6, Tnfα,* and *Ifnγ,* in the brain, spinal cord, and spleen, using qRT-PCR. Fold gene expression was normalized to *Gapdh* and *β-Actin* and error bars represent the SEM of triplicate measures (**p ≤ 0.01). **i** Graphical representation of Treg (CD4 + CD25 + FOXP3 +) and Th17 (CD4 + IL-17A +) cells in the blood, spleen, brain, and spinal cord as determined by flow cytometry (**p ≤ 0.01). Treg cell numbers went down, but Th17 went up in the blood and spleen, and both of these cells were significantly increased in the CNS of EAE animals. Transplanted cells significantly increased Treg but significantly reduced Th17 cells in the blood and spleen, and both of these cells were significantly reduced in the CNS of EAE animals. **j** Splenocytes isolated from control and EAE mice were cultured and treated with MOG_35–55_. After 72 h following MOG activation, the cultures were treated with the MSCs and NSCs, and splenocyte proliferation was determined using BrdU assay

EAE induction mediates T cell activation resulting in increased infiltrates in the CNS by permeation of the BBB and progression of disease pathogenesis [20]. As expected, the brain and spinal cord (Fig. 4a, b) showed the presence of cell infiltrates in EAE animals but not in healthy controls. The number of infiltrates in both the brain and spinal cord was significantly decreased in animals treated with MSCs and NSCs; however, NSCs exhibited a greater reduction in infiltrates in the cortex region. Altogether, these results suggest that transplanted NSCs reduced cell infiltrates more significantly.

To investigate the effect of transplanted cells on the inflammatory response in EAE mice and characterize the infiltrates observed in the H&E results, we analyzed the expression of CD45, CD68, and CD3E markers, representing leukocytes, macrophages, and T cells, respectively. The results depicted in Fig. 4c, d, e show high expression of these markers in the brain and spinal cord of EAE animals but not in healthy controls. Importantly, NSCs were more effective in reducing the expression of all three markers in the spinal cord.

It is well known that EAE and MS are caused by an inflammatory response [21]. Therefore, transcriptional analysis of the brain, spinal cord, and spleen tissues was performed to determine the effects of transplanted cells on the expression of inflammatory genes. As expected, expression of the inflammatory markers, *Cd3e*, *Il-17a*, *Il-2*, *Il-1β*, *Il*-6, *Tnfα*, and *Ifnγ*, was increased in the CNS and spleen in EAE animals compared to the healthy controls (Fig. 4f–h). When EAE animals were transplanted with cells, the expression of inflammatory genes was significantly reduced in the CNS of MSC and NSC-treated EAE animals. The expression of inflammatory genes was also reduced in the spleen (Fig. 4h). In general, NSCs displayed a greater modulatory effect on inflammatory gene expression than MSCs. Overall, NSCs exerted a greater anti-inflammatory response, potentially by reducing the number of pro-inflammatory cells, macrophages, leukocytes, and T cells.

### Immunomodulation of Treg and Th17 by transplanted cells

Treg (CD4 + /CD25 + /FOXP3 +) and Th17 (CD4 + /IL17A +) are the main effector cells of cell-mediated immune response in EAE and MS [20]. We investigated the levels of these cells in the blood, spleen, and CNS by flow cytometry. Treg cells were significantly reduced to 7.7% and 8.0% in EAE animals from 34.1% and 27.5% in healthy controls in both the blood and spleen (Fig. 4i). In contrast, Treg cells were substantially increased in EAE animals transplanted with cells in the blood and spleen. In EAE animals treated with MSCs, Treg cells were 17.4% and 22.5% in the blood and spleen, respectively, whereas in NSC-treated animals, Treg cell levels were 27.3% and 25.4% in the blood and spleen, respectively. In conclusion, Treg cell numbers increased in animals treated with both MSCs and NSCs; however, more significant improvements in the restoration of Treg levels in the blood were observed in the case of NSCs than MSCs. In the CNS of EAE animals, Treg levels were 10.5% and 12.0%, respectively. Although cell transplantation reduced Treg cells in the brain, they were more significantly reduced to 5.7% and 3.9% in the spinal cord of EAE animals treated with MSCs and NSCs, respectively. Taken together, NSCs showed greater efficacy than MSCs in reducing Treg cells in the spinal cord in EAE animals. It is known that Treg cells increase during the chronic late scores of EAE [22]. Therefore, it is plausible that reduced Treg levels in animals treated with cells could improve disease symptoms.

As expected, Th17 cells were significantly increased from 10.3 and 6.9% in the blood and spleen of healthy controls to 15.3% and 13.7% in EAE animals, respectively. When treated with MSCs, Th17 cell levels were not significantly affected in the blood (14.9%) but were reduced to normal levels (10.1%) in the spleen (Fig. 4i). Importantly, NSC transplantation reduced Th17 to levels similar to the healthy control animals. The brain and spinal cord of EAE animals had significantly higher levels of Th17 cells (22.5% and 30.5%, respectively) compared to healthy control animals (1.6% and 0.9%, respectively). Upon MSC transplantation, Th17 cells were significantly reduced in the brain and spinal cord (12.6% and 20.3%, respectively). On the other hand, NSC transplantation had a more prominent effect on reducing Th17 cells in both the brain and spinal cord (11.5% and 13.7%, respectively) in EAE animals. Clearly, NSCs showed greater immunomodulatory effects in EAE mice.

It is conceivable that the transplanted cells could ablate or regulate CNS autoreactive T cells. To investigate this possibility, we evaluated MOG-activated splenocyte proliferation treated with MSCs and NSCs ex vivo. The BrdU analysis showed T cell proliferation stimulated with MOG in both control and EAE splenocytes but had an insignificant effect upon treatment with MSCs and NSCs (Fig. 4j).

### Cell transplantation reduced reactive astrocyte and gliosis in CNS

**Fig. 5:**
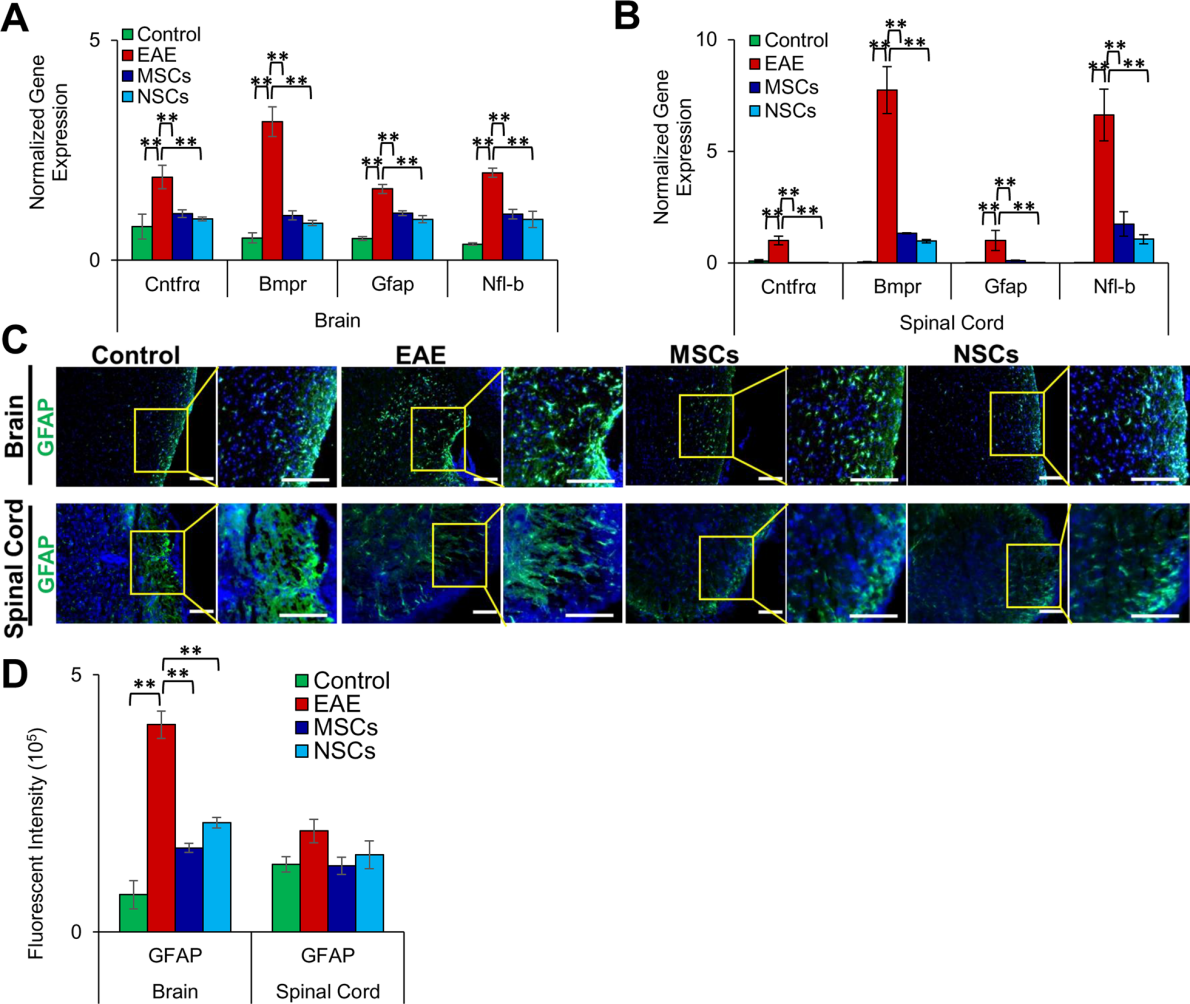
Suppression of gliosis by transplanted cells. **a**, **b** Transcription of genes, *Cntfrα, Bmpr, Gfap,* and *Nfl-b,* involved in gliosis in the brain and spinal cord, using qRT-PCR. Fold gene expression was normalized to *Gapdh* and *β-Actin* and error bars represent the SEM of triplicate measures (**p ≤ 0.01). Expression of gliosis genes was significantly upregulated in EAE mice but reduced significantly in treated animals. **c** Immunohistochemical staining of paraffin sections of the brain and spinal cord with antibody astrogliosis marker, GFAP. The inserts (on the right) were magnified and overexposed to enhance contrast. Scale bars represent 50 µm scale bars (magnification: 40 ×). **d** Quantification of fluorescent intensity of GFAP in the brain and spinal cord sections depicted in **c**, respectively (**p ≤ 0.01). GFAP levels significantly increased in EAE mice. However, they were reduced considerably in transplanted animals

Upon induction of EAE, the expression of GFAP is increased (Fig. 5a–d), due to the activation of astrocytes in response to gliosis [23]. We first examined the expression of *Cntfrα, Bmpr, Gfap,* and *Nfl-b* associated with astrogliosis [24, 25] via qRT-PCR. As expected, these genes were increased in the CNS of EAE animals (Fig. 5a, b). However, the expression of the gliosis-associated genes significantly decreased in the CNS tissues of EAE animals transplanted with cells. Specifically, EAE animals transplanted with NSCs displayed a greater effect in reducing these genes in the spinal cord. We then examined GFAP expression at a protein level through immunostaining of CNS tissue sections. Overall, GFAP expression was decreased in the CNS of EAE animals transplanted with cells, but was only significant in the brain and not in the spinal cord (Fig. 5c, d). Transplantation of NSCs promoted a greater reduction in gliosis in the EAE animals.

### Cell treatment promoted remyelination

**Fig. 6:**
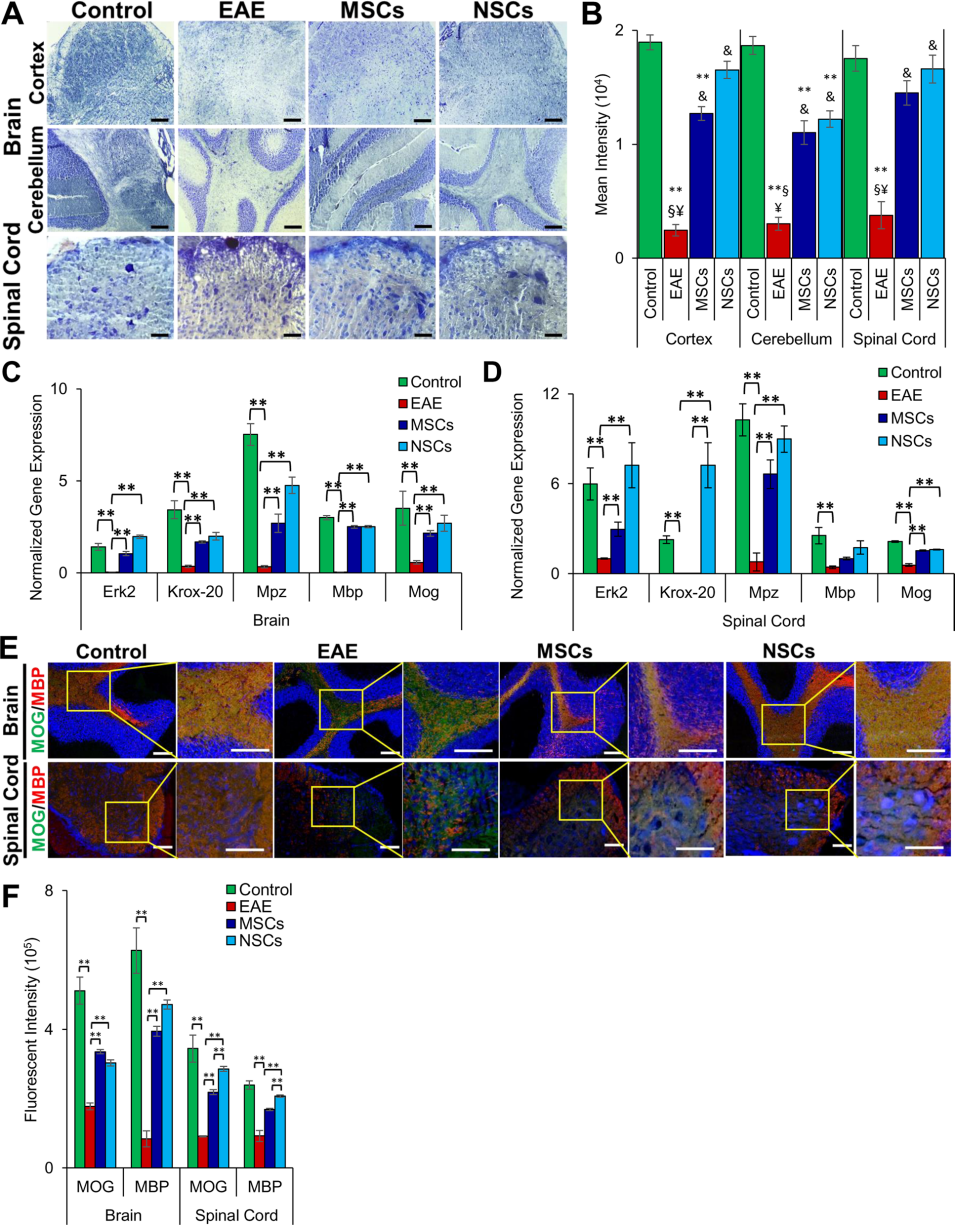
Cell transplantation resulted in improvement in myelination of CNS. **a** Analysis of paraffin sections of the brain and spinal cord stained with LFB to detect myelination. Scale bars represent 50 μm (magnification: 40 ×). **b** Quantification of LFB stain intensity of paraffin sections shown in **a**. Symbols, **, &, §, and ¥ indicate significant difference at *p* ≤ 0.01 between all experimental conditions: healthy control, EAE, EAE + MSCs, and EAE + NSCs. LFB stain intensity was significantly improved in the cortex, cerebellum, and spinal cord in transplanted EAE animals. **c**, **d** Transcription of myelination markers, Erk*2, Krox-20, Mpz, Mbp,* and *Mog* in the brain and spinal cord*.* Fold gene expression was normalized to *Gapdh* and *β-Actin* and error bars represent the SEM of triplicate measures (**p ≤ 0.01). **e** Immunohistochemical staining of myelin proteins, MBP and MOG, in the brain and spinal cord paraffin sections. The inserts (on the right) were magnified and overexposed to enhance contrast. Scale bars represent 50 µm scale bars (magnification: 40 ×). **f** Quantification of fluorescent intensity of myelin proteins in the brain and spinal cord sections depicted in **e** (**p ≤ 0.01)

LFB staining of CNS tissue sections was performed to investigate the effect of transplanted cells on the degree of myelination. While LFB stain intensity was reduced in EAE mice, it was significantly improved in transplanted animals (Fig. 6a, b). The staining intensity was greater in NSC-treated animals compared to those treated with MSCs. An increase in the staining intensity in the cortex and spinal cord suggests that transplanted cells induced endogenous remyelination.

To investigate the cause of increased myelin, we then examined myelin-associated gene expression in the CNS tissue by qRT-PCR. The expression of all tested myelination-associated genes, *Erk2*, *Krox-20*, *Mpz*, *Mbp*, and *Mog*, was significantly upregulated in the CNS of the transplanted EAE mice (Fig. 6c, d), except *Krox-20*, and *Mbp* was not upregulated in the spinal cord in the case of MSCs. We validated these results by performing immunostaining of myelin-associated proteins, MBP and MOG. The expression of MBP and MOG in the brain of EAE animals (Fig. 6e, f) was significantly increased upon transplantation of MSCs and NSCs. The trend in the expression of MBP and MOG in the spinal cord of EAE animals treated with cells was similar to the brain, while MBP expression was greater in EAE animals treated with NSCs in the spinal cord. These results suggest that transplanted NSCs showed increased remyelination in the CNS in EAE animals than MSCs.

### Cell transplantation upregulated genes involved in neuroprotection

**Fig. 7:**
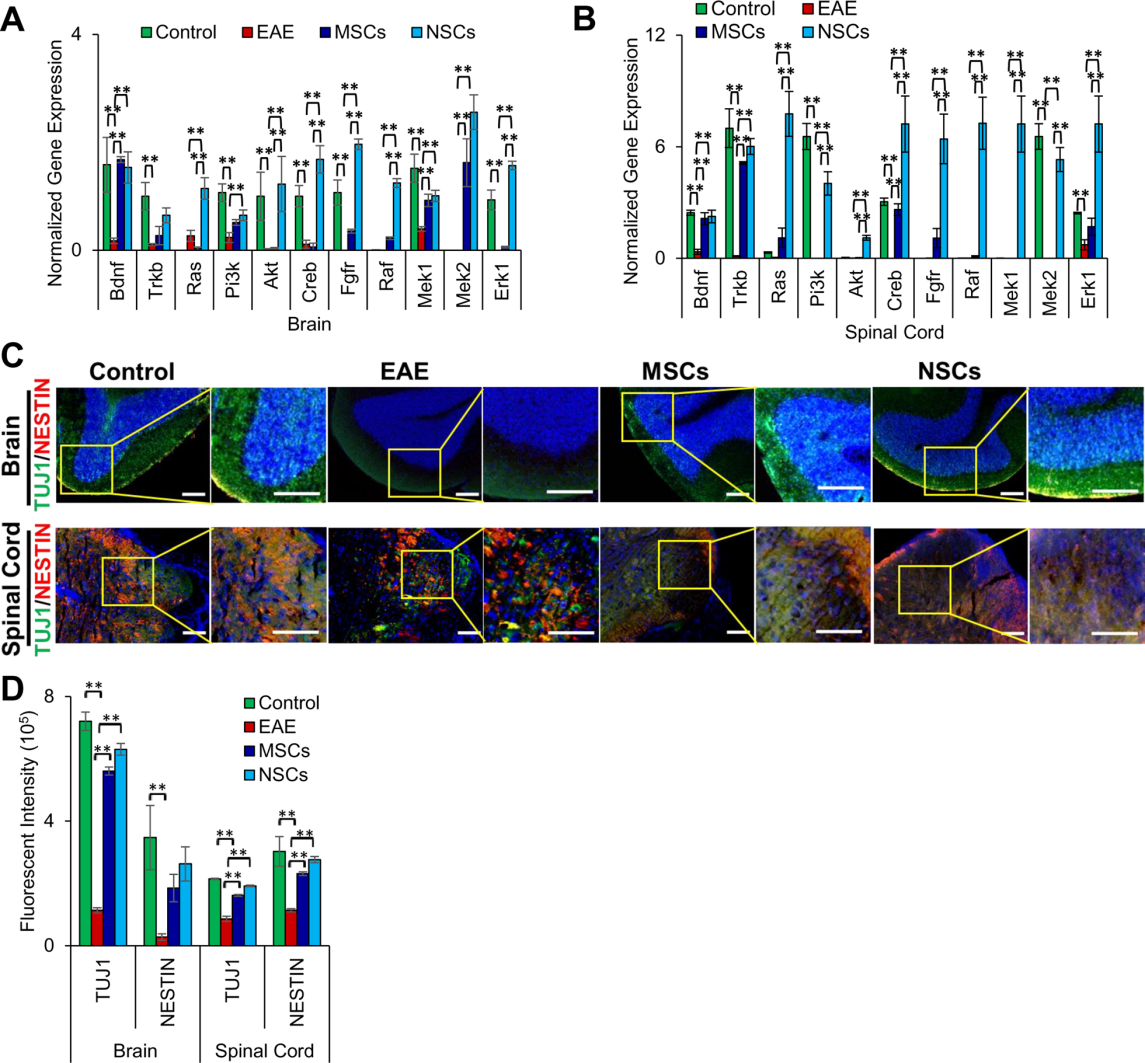
Cell transplantation upregulated neuroprotection markers. **a**, **b** Transcription of neuroprotection markers, *Bdnf, Tkrb, Ras, Pi3k, Akt, Creb, Fgfr, Raf, Mek1, Mek2,* and *Erk1*, in the brain and spinal cord, respectively, analyzed using qRT-PCR. Fold gene expression was normalized to *Gapdh* and *β-Actin* and error bars represent the SEM of triplicate measures (**p ≤ 0.01). All of the tested markers were increased to normal or higher levels in NSC, but not MSC -transplanted animals compared to the healthy controls. **c** Immunohistochemical staining of paraffin sections of the brain and spinal cord using antibodies against neural proteins, TUJ1 and NESTIN. The inserts (on the right) were magnified and overexposed to enhance contrast. Scale bars represent 50 µm scale bars (magnification: 40 ×). **d** Quantification of fluorescent intensity of immunostaining in the brain and spinal cord sections depicted in **c**. (**p ≤ 0.01). Expression of the neural proteins was more significantly increased in transplanted NSCs than MSCs in EAE animals

Neurotrophic factors support the growth, survival, and differentiation of both developing and mature neurons in the CNS [26]. It has been suggested that the neurotrophic factor BDNF plays an important role in axon protection during autoimmune demyelination of the CNS [27]. Our analysis showed an extensive decrease in expression of all the tested genes, *Bdnf*, *Trkb*, *Ras, Pi3k, Akt, Creb, Fgfr, Raf, Mek1, Mek2,* and *Erk1,* associated with neuroprotection in EAE mice (Fig. 7a, b). When EAE mice were transplanted with cells, the expression of all genes (except *Trkb* in the brain) was significantly increased, and NSCs had a greater effect than MSCs. In most cases, the expression was near or higher than the healthy control levels in both the brain and spinal cord. These results clearly show that NSCs provided greater neuroprotection than MSCs.

We next examined the effect of transplanted cells on the expression of neural proteins. We observed a significant decrease in the expression of TUJ1 and NESTIN in the brain of EAE animals (Fig. 7c, d). When EAE animals were transplanted with cells, TUJ1 but not NESTIN expression was significantly improved and was near-normal levels in the brain. In contrast, the expression of both TUJ1 and NESTIN was improved significantly and was near normal levels in the spinal cord. These results suggest cell transplantation also promoted neural development.

### Differentiation of transplanted cells in CNS

**Fig. 8:**
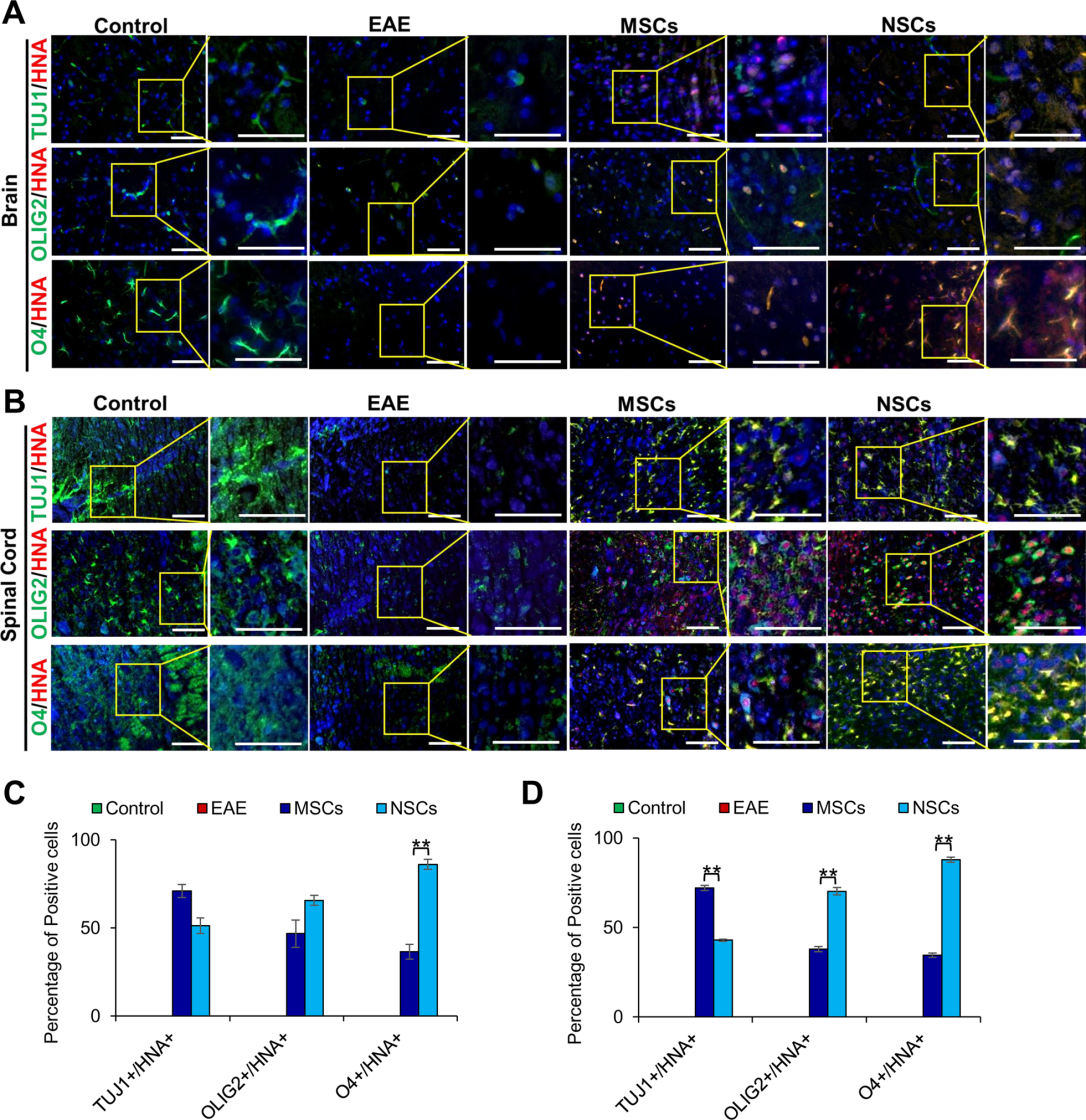
Transplanted cells expressed neural and oligodendrocyte proteins that co-localized with HNA in vivo. **a**, **b** Immunohistochemical staining of paraffin sections of the brain and spinal cord with antibodies against cell-specific proteins, TUJ1 (NSCs), OLIG1 (oligodendrocyte progenitor cells), and O4 (ODCs), and counterstained with antibody against HNA. The inserts (on the right) were magnified and overexposed to enhance contrast. Scale bars represent 50 µm scale bars (magnification: 40 ×). **c**, **d** Percentage of TUJ1^+^/HNA^+^, OLIG2^+^/HNA^+^, and O4^+^/HNA^+^ cells in the brain and spinal cord sections shown in **a** and **b**, respectively (**p ≤ 0.01). TUJ1 staining intensity was greater in both the brain and spinal cord in the case of MSCs, and OLIG2 and O4 staining intensities were greater in the case of NSCs

To investigate the fate of transplanted cells, CNS tissue sections were co-stained with cell-specific antibodies, TUJ1, OLIG2, and O4, against neural cells, oligodendrocyte progenitors, and ODCs, respectively, as well as HNA, and visualized by fluorescent microscopy. The results depicted in Fig. 8a, b showed HNA-positive cells were found in the CNS of the transplanted EAE animals. The results in Fig. 8a–d showed that while the cell-specific markers decreased in EAE animals, their expression was co-localized with HNA and significantly improved in transplanted cells. Of the HNA-positive cells, 70.9% and 72.0% cells were positive for TUJ1 in the brain and spinal cord of MSC injected animals, respectively, whereas only 51.3% and 42.9% of the HNA-positive cells expressed TUJ1 in the brain and spinal cord of the NSC-treated animals, respectively. Further analysis revealed that 46.7% and 37.9% of HNA-positive cells were also OLIG2 positive while 36.5% and 34.4% of HNA-positive cells expressed O4 in the brain and spinal cord in MSC-treated animals, respectively. In the case of NSC-treated animals, 65.6% and 86% of HNA-positive cells were OLIG2 positive in the brain and spinal cord. A higher number of HNA-positive cells (70.2% and 87.8%) were positive for O4 in the brain and spinal cord, respectively, in NSC-treated animals. Evidently, a significantly higher number of transplanted cells differentiated towards the ODC lineage in the case of NSCs than MSCs. These results suggest that transplanted cells promoted neural development and regeneration.

### Cell transplantation results in the upregulation of neurogenesis genes

**Fig. 9:**
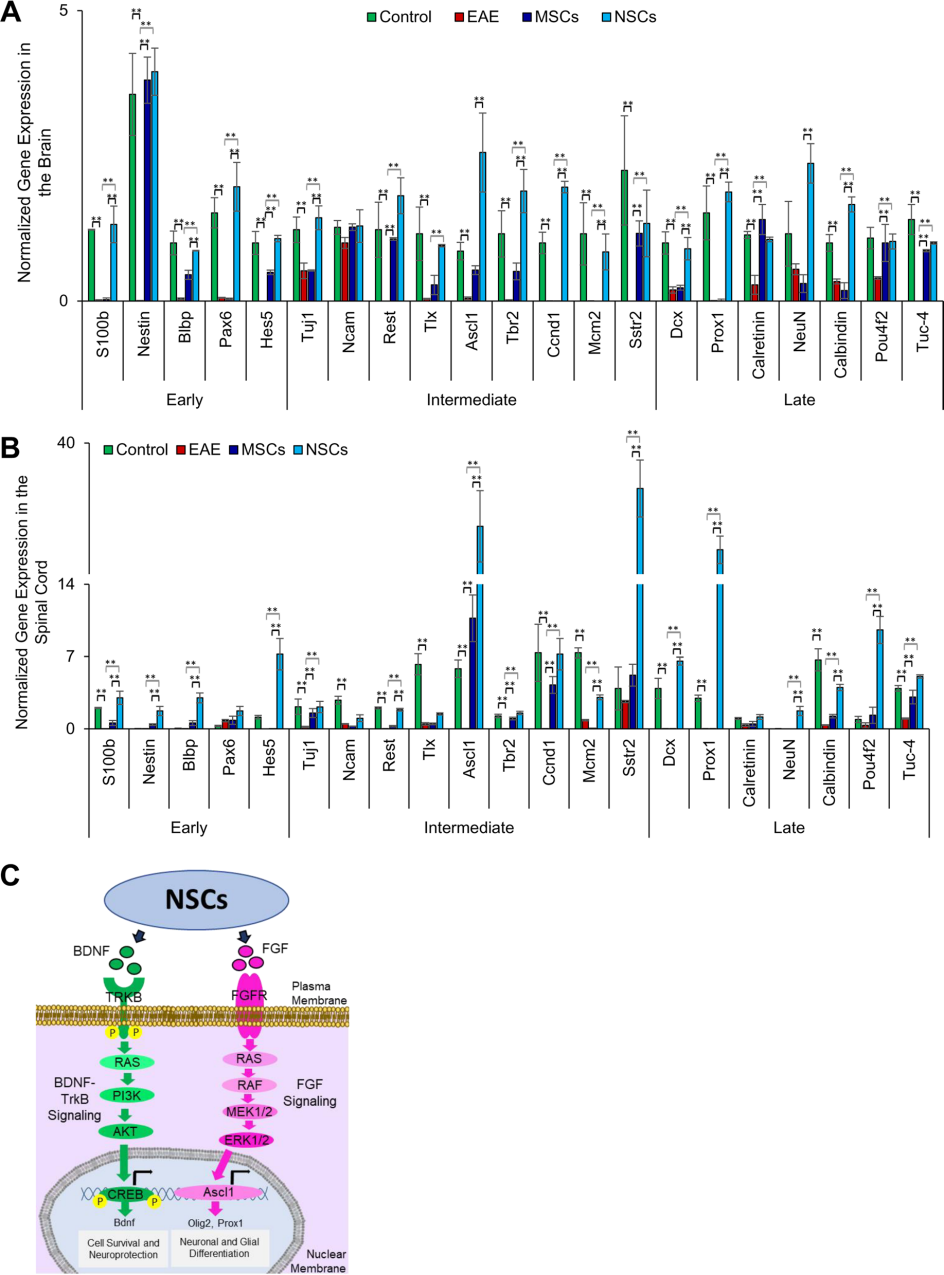
Cell transplantation resulted in the upregulation of neurogenesis markers in the CNS. **a**, **b** Determination of expression of genes associated with various stages of neurogenesis, early (*S100b, Nestin, Blbp, Pax6, Hes5*), intermediate (*Tuj1, Ncam, Rest, Tlx, Ascl1, Tbr2, Ccnd1, Mcm2, Sstr2*) and late (*Dcx, Prox1, Calretinin, NeuN, Calbindin, Pou4f2, Tuc-4*) using qRT-PCR. Fold gene expression was normalized to mouse *Gapdh* and *β-Actin* and error bars represent the SEM of triplicate measures (**p ≤ 0.01). Expression of genes representing all stages of neurogenesis was greater in the case of NSCs than MSCs, but it was more pronounced in the middle and late stages of neurogenesis. **c** Proposed molecular mechanism of cell survival/neuroprotection and differentiation promoted by NSCs

Recent studies have demonstrated that neurogenesis genes are downregulated in the CNS tissues of EAE animals [28]. Since cell fate analysis indicated in vivo differentiation of transplanted cells (Fig. 8), we performed the transcriptional analysis of genes known to promote neuron development and growth. As anticipated, neurogenesis genes, early (*S100b, Nestin, Blbp, Pax6,* and *Hes5)*, intermediate (*Tuj1, Ncam, Rest, Tlx, Ascl1, Tbr2, Ccnd1, Mcm2,* and *Sstr2)*, and late (*Dcx, Prox1, Calretinin, NeuN. Calbindin, Pou4f2,* and *Tuc-4)*, were downregulated in both the brain and spinal cord of EAE animals. However, transplanted cells rescued the expression of these genes in the CNS (Fig. 9a, b). NSCs had a significantly greater expression of the neurogenesis genes in both the brain and spinal cord when compared to animals transplanted with MSCs. Taken together, NSCs resulted in a greater improvement in promoting neurogenesis via the upregulation of *Ascl1, Sstr2, Prox1, Pou4f2,* and *Dcx* in the CNS tissues.

Our results showed that expression of *Ras, Pi3k, Akt,* and *Creb* were downregulated in EAE animals but increased in NSC-transplanted animals (Fig. 7). This may suggest that the BDNF-TrkB signaling pathway, which plays a crucial role in promoting cell survival and neuroprotection in vivo [29, 30], is at least partially responsible for functional neural recovery in transplanted animals. Furthermore, several genes, including *Fgfr, Raf, Mek1/2, Erk1/2,* and *Olig2* as well as *Ascl1* and *Prox1*, which are associated with the FGF signaling pathway and involved in neuronal and glial differentiation [29, 31], were down-regulated in EAE mice, but their expression was significantly increased in transplanted NSCs. Our results suggest that NSC transplantation modulated the BDNF-TrkB and FGF signaling pathways, as illustrated in Fig. 9c.

## Discussion

In the current study, we used highly proliferative and naïve primitive MSCs isolated from a specific region of the umbilical cord tissue [7, 8] to differentiate them into a large amount of NSCs required for animal studies. Primitive MSC-derived NSCs expressed known neural markers, including NESTIN, TUJ1, VIMENTIN, and PAX6 [32]. Although published reports indicate expression of NESTIN [33] and VIMENTIN [34] by MSCs, our results showed that MSCs exhibited significantly lower expression of these markers. Further differentiation of NSCs yielded cells with ODC characteristics as they expressed typical markers, OLIG2, SOX10, O4, MBP, and MOG [35].

EAE disease progression and symptoms in the established mouse model followed a typical pattern and were scored according to published reports [16]. EAE was induced 10–11 days following MOG immunization, and clinical symptoms got progressively worse, as predicted. In addition, weight was found to be negatively correlated with disease symptoms, with an observed decrease in weight in EAE mice. Animals at score 1 and 2 of EAE were randomly transplanted with labeled MSCs and NSCs. PKH26 lipophilic tracer was used to label the cells, which has been successfully used to track cells in vivo for more than 8 weeks, as reported in our previous studies [17, 36]. Following cell transplantation, animals showed significant improvement in the clinical symptoms. The symptoms progressively and reproducibly improved in EAE disease score 1 animals than score 2 animals. Although MSCs slowed and halted the disease progression, results of NSC transplantation were even more encouraging at disease score 1. NSCs slowed and reversed the disease process as the neurobehavior of transplanted EAE animals improved to near-normal levels. In brief, NSC treatment proved to be more efficacious based on the clinical symptoms, particularly in EAE disease score 1. Therefore, post-transplantation analysis was focused on EAE disease score 1 animals.

Our cell tracking data showed that the majority of the transplanted labeled cells were localized in the blood, spleen, and CNS in EAE animals. This is consistent with previous studies where MSCs were transplanted in EAE mice and were found to home towards the spinal cord tissue [37]. However, in our study, a significant number of injected MSCs and NSCs were found in the spleen, brain, and spinal cord, demonstrating their homing potential to migrate to multiple tissues typically affected by EAE. Interestingly, significantly more transplanted NSCs than MSCs were observed in the spinal cord. Although labeled cells transplanted in healthy animals were not found in CNS, they were able to permeate the BBB in diseased animals as BBB is compromised in EAE [20].

In EAE, activated T cells travel through the BBB, leading to increased cell infiltrates in the CNS [20]. We also observed a significant increase in cell infiltrates in the brain and spinal cord of EAE animals. However, in EAE animals transplanted with cells, the number of infiltrates in the CNS decreased significantly. Previously, adipose MSCs have been shown to inhibit cell infiltrates in the EAE animal model [38]. However, in our study, NSCs displayed a greater effect than MSCs in reducing the number of infiltrates in the CNS. Although NSCs derived from various sources have modulated immune response in EAE animal models [39–42], this is the first report where NSCs derived from primitive MSCs were used and showed greater effectiveness than MSCs. As anticipated, our immunostaining results showed that infiltrates comprised leukocytes, macrophages, and T cells [39]. Since EAE induces a pro-inflammatory response, the results of this study showed that transplanted cells modulated the immune response, significantly reducing the expression of cytokines, *Cd3e*, *Il-17a*, *Il-2*, *Il-1β*, *Il*-6, *Tnfα*, and *Ifnγ*, in the CNS and spleen. In EAE, IL-6 aggravates clinical symptoms and spinal cord pathology mainly by promoting pathogenic Th17 cells, which initiate and perpetuate inflammation and demyelination [40, 41]. Our results showed that cell transplantation effectively counters the induction of *Il-6* gene expression in the spleen and the CNS tissues. This suggests that transplanted cells exerted an anti-inflammatory response.

Studies have shown that Treg and Th17 play an important role in the development of EAE and the immune response correlated with the disease [42–44]. Treg maintains cell immune tolerance, whereas Th17 mediates the inflammatory response under abnormal conditions such as during an injury [43]. As expected, we observed an imbalance between Treg and Th17 in EAE animals. At the onset of EAE, the levels of Treg decreased and Th17 increased in the blood and spleen, but both increased in the CNS. However, after NSC transplantation, the imbalance was corrected, and the levels of Treg and Th17 resembled those found in the blood and spleen of the healthy controls, whereas levels of both Treg and Th17 were reduced in the CNS of animals transplanted with cells. The underlying mechanism for restoring Treg/Th17 levels could be due to the transplanted cells modulating the function of T cells by inhibiting the production of TNFα and IFNγ as shown in our study and reported previously [45–47]. Although MOG induced a significant increase in the activation of splenocytes ex vivo [48], both MSCs and NSCs had an insignificant effect on the proliferation of splenocytes. These findings suggest that the transplanted cells did not have a tolerizing effect of ablating or regulating CNS autoreactive T cells.

In order to elucidate the mechanism that might explain the therapeutic effects of MSCs and NSCs in EAE mice, we investigated gliosis in the CNS. It is known that in chronic EAE, lesions become inactive resulting in accumulation of reactive astrocyte (GFAP+) filaments in the CNS [49]. We found that astrogliosis gene, *Gfap*, was upregulated in EAE mice as well as GFAP+ mouse cells, which were integrated into the brain and grey matter of the spinal cord. Upon cell transplantation, a significant decrease in the expression of astrogliosis genes, *Cntfrα, Bmpr, Gfap,* and *Nfl-b,* as well as a recession of GFAP+ cells in the grey matter was in agreement with previous reports [45, 50, 51].

Since cell transplantation reduced infiltrates, induced anti-inflammatory response, and inhibited gliosis, they were likely to promote remyelination as reported previously [52, 53]. Our approach significantly restored the lost myelin in EAE animals, particularly in the case of transplanted NSCs. Loss of ODCs and activated T cells in MS/EAE contributes to demyelination of neurons [54]. Our results showed significant upregulation of myelin associated genes, *Erk2, Krox-20, Mpz, Mbp,* and *Mog,* and proteins, MBP and MOG, suggesting restoration of ODCs resulting in significant improvement in myelination observed in animals transplanted with cells, particularly with NSCs.

Therefore, we investigated whether transplanted cells themselves differentiate into the glial lineage in vivo. Analysis of CNS tissues showed co-localization of expression of HNA and genes, TUJ1, as well as OLIG2 and O4, which are key factors in oligodendrogenesis [55, 56]. Interestingly, MSCs displayed an upregulation of TUJ1 providing evidence that the MSCs had entered into the early stages of neural differentiation, whereas the upregulation of OLIG2 and O4 in the case of NSC transplantation suggested that they differentiated towards the ODC lineage. Therefore, it is plausible to suggest that NSCs have potential to differentiate in vivo to replace, regenerate, and repair the lost cells due to EAE.

Typically, NSCs reside in the hippocampus hilus and subventricular zone, which promote proliferation and migration in different diseases [28]. Moreover, these cells have the potential to differentiate to produce mature astrocytes and neurons through the enhancement of neurogenesis [57]. However, the therapeutic potential of self-neurogenesis in the mature CNS is very limited due to the sharp decline of this process after birth [28, 58]. In the current study, results showed that there was an overall increase in the expression of neurogenesis-specific genes in the CNS tissues in NSC-transplanted animals. Additionally, NSC-transplanted animals displayed the greatest upregulation of *Ascl1*, *Sstr2*, and *Prox1* in the spinal cord, which is known to promote neuronal and ODC differentiation [29, 31]. Presumably, these factors contributed to the greater efficacy of NSCs for neurogenesis and the reversal of EAE symptoms by preserving and regenerating the neurons. The fact that the expression of these factors is improved more significantly in animals transplanted with NSCs and that the animals recovered to near normal conditions suggests the use of NSC therapy in MS patients would be more effective than using MSCs.

One of the possible reasons NSCs are more effective is their ability to express neurotrophins, such as BDNF, a regulator of adult neurogenesis [59]. Studies have revealed that TrkB has the greatest relative affinity for BDNF and is expressed on a large proportion of stem cells during differentiation and maturation towards the neural lineages [59]. When BDNF binds to TrkB, a downstream cascade effect is triggered by activating RAS, PI3K, and AKT. This activation leads to upregulation of the nuclear gene transcription of *CREB* [30]. Studies have shown that when *CREB* is upregulated in differentiating neurons, it will enhance neuronal proliferation and cell survival [60]. In this study, we observed the upregulation of markers associated with BNDF-TrkB signaling which resulted in the increased expression of *Ras*, *Pi3k*, and *Akt* as well as *Creb* and *Bdnf* in animals treated with NSCs. Although EAE animals transplanted with MSCs displayed a significant increase of *Bdnf* and *Trkb*, downstream gene expression was insignificant compared to untreated EAE animals. Therefore, NSCs displayed a greater effect in promoting proliferation and neuron maturation via activation of the BDNF-TrkB pathway through BDNF stimulation.

FGF has been found to be a potent modulator of the proliferation and differentiation of neural progenitor cells within the CNS [61]. Additionally, FGF signaling is important for the regulation of neurogenesis in the CNS [62]. A study revealed that upregulation of FGF signaling led to an increase in the neural differentiation process [63]. When FGF binds to the FGF receptor, this triggers the intracellular pathway that leads to the cascade activation of RAS, RAF, MEK1, MEK2, ERK1, and ERK2 [64]. *Ascl1* is then transcribed due to this cascade effect leading to neuronal and ODC differentiation [65]. Our results showed a significant upregulation of *Fgfr*, *Ras*, *Raf*, *Mek1*, *Mek2*, *Erk1*, and *Erk2,* as well as *Ascl1* and *Prox1*, particularly in NSC-transplanted animals with the greatest effect in the spinal cord. Significantly, higher expression of *Ascl1* in animals transplanted with NSCs suggests that the cells promoted neuronal differentiation as well. Altogether, our results indicate that BDNF-TrkB and FGF signaling might play an important role in promoting proliferation, cell survival, and neuronal and glial differentiation observed when transplanted NSCs in an EAE animal.

## Conclusion

In brief, this study demonstrated the potential of NSCs to induce anti-inflammatory responses and provide neuroprotection and promote endogenous neurogenesis resulting in the reversal of EAE clinical symptoms and repair of the damaged CNS. Although there have been a number of clinical trials utilizing autologous bone marrow MSCs and umbilical cord-derived MSCs, the results are not always conclusive [66–68]. Furthermore, most of these studies were limited to phase I and II trials due to the limited availability of cells. Two clinical trials used human fetal-derived NSCs [69]; however, results of these phase I/II studies have not been reported. Furthermore, NSCs were isolated from their fetal tissue, and their use could pose ethical and moral concerns [70]. The results of our study are highly promising in reversing the disease process and improving the EAE pathology of CNS, particularly in the case of NSCs. Significantly, since primitive MSCs used here are highly proliferative and can be maintained for many passages without losing their differentiation potential, they can be rapidly amplified and differentiated into NSCs in the amounts required for large animals and clinical studies. This has been one of the major impediments for conducting phase III and IV using MSCs or their derivatives. Further studies should be pursued to determine if higher or multiple doses of cells injected at advanced/chronic stages of EAE could also effectively reverse the EAE disease process. It would also be interesting to know if cell treatment at the time of MOG injection could block the induction of EAE in mice. Further studies are warranted to investigate the modulation of Treg cells by NSCs. Additional studies for elucidating the cellular and molecular mechanism and associated signaling pathways for functional neuronal recovery would be helpful. Nonetheless, our study provided proof of concept and basis for investigating the efficacy of primitive MSC-derived NSCs to treat MS in clinical trials.

## Supplementary Information

**Additional file 1**. List of primer sequences used in qRT-PCR.

**Additional file 2**. MOG-induced EAE score scale and clinical symptoms.

**Additional file 3**. Detailed analysis of cell transplantation on EAE clinical symptoms and weight. (a) Graphical representation of the day the cells were transplanted for each EAE group. (b) Clinical parameters of the EAE mice prior to and after treatment with primitive MSCs or NSCs.

## Data Availability

All data generated or analyzed during this study are included in this published article and its supplementary information files.

## Abbreviations

BBB: Blood–brain barrier
bFGF: Basic fibroblast growth factor
CNS: Central nervous system
EAE: Experimental autoimmune encephalomyelitis
EGF: Epidermal growth factor
FBS: Fetal bovine serum
GM: Growth medium
GvHD: Graft verse host disease
H&E: Hematoxylin and eosin
i.p: Intraperitoneally
IACUC: Institutional Animal Care and Use Committee
IBC: Institutional Biosafety Committee
LFB: Luxol fast blue
ODCs: Oligodendrocytes cells
MHC: Major histocompatibility complex
MOG: Myelin oligodendrocyte glycoprotein
MS: Multiple sclerosis
MSCs: Mesenchymal stem cells
NSCs: Neural stem cells
qRT-PCR: Quantitative reverse transcriptase polymerase chain reaction
SEM: Standard error of the mean
TBST: TBS 1X containing 0.1% Tween-20
