# Supplementary figures and images for: Neural stem cells derived from primitive mesenchymal stem cells reversed disease symptoms and promoted neurogenesis in an experimental autoimmune encephalomyelitis mouse model of multiple sclerosis
Source: Stem Cell Res Ther. 2021 Sep 9;12:499. doi: 10.1186/s13287-021-02563-8 (PMC8427882; doi:10.1186/s13287-021-02563-8)

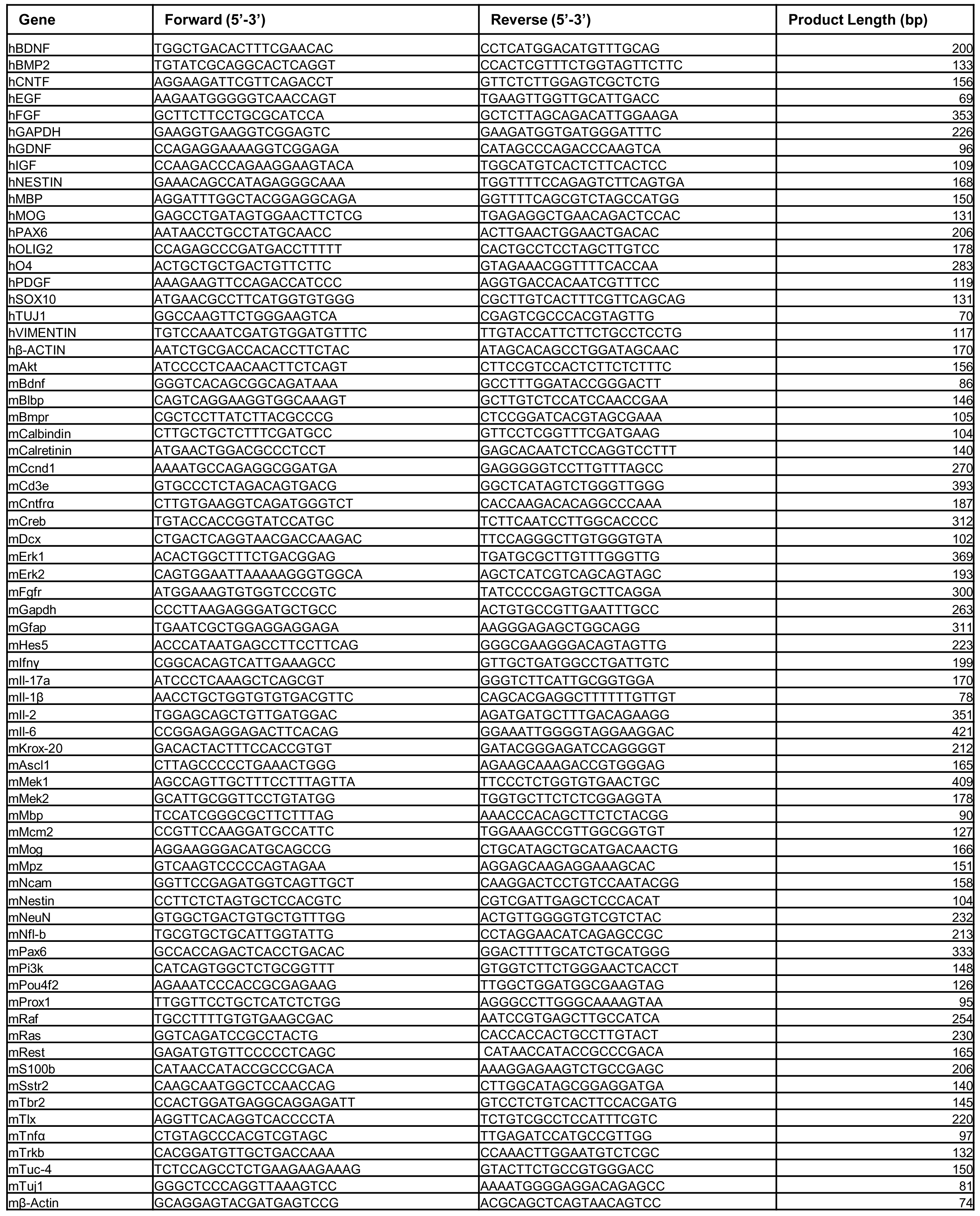

Supplement: Supplementary file 1 — Additional file 1. List of primer sequences used in qRT-PCR. [file 13287_2021_2563_MOESM1_ESM.tif]

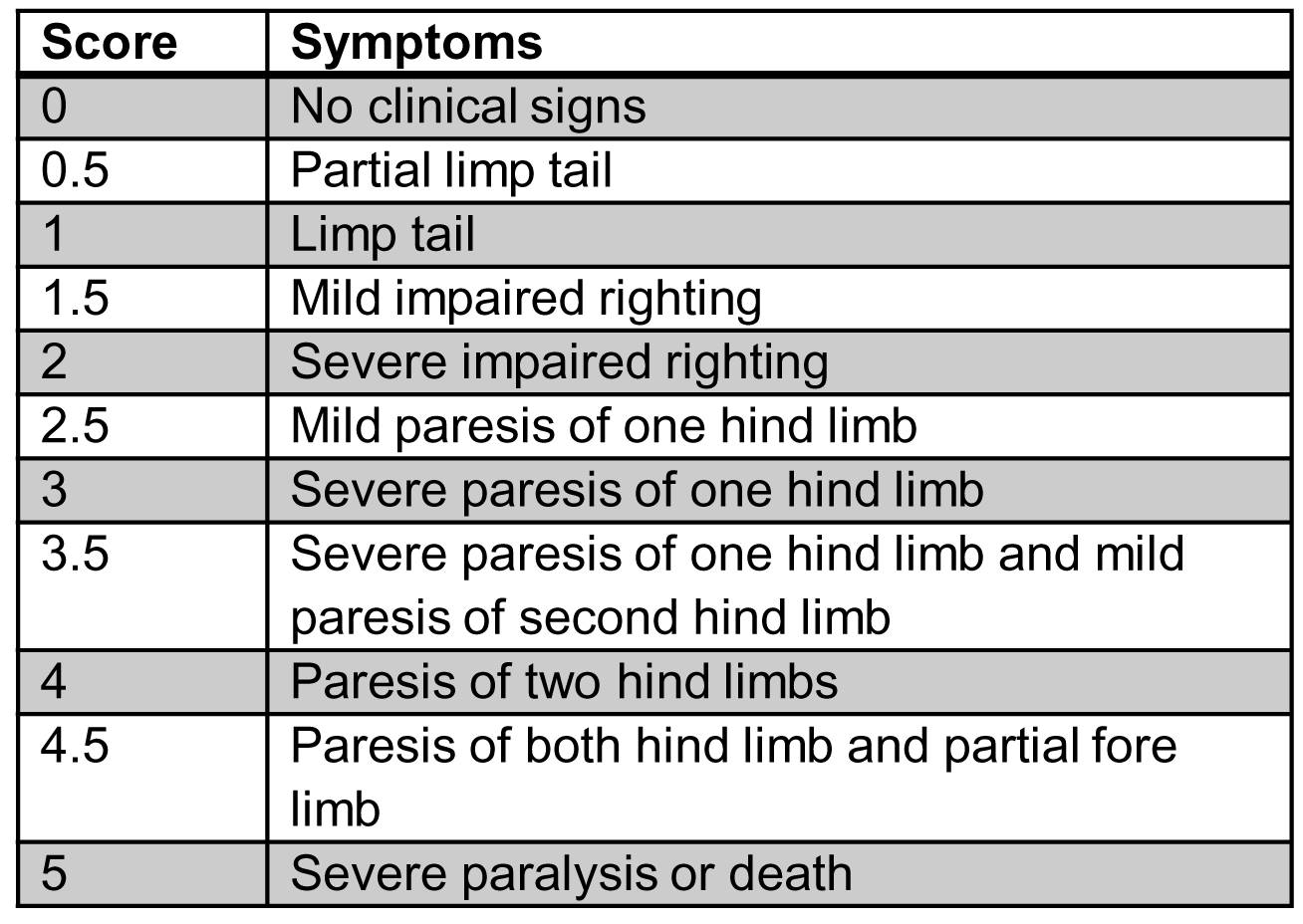

Supplement: Supplementary file 2 — Additional file 2. MOG-induced EAE score scale and clinical symptoms. [file 13287_2021_2563_MOESM2_ESM.tif]

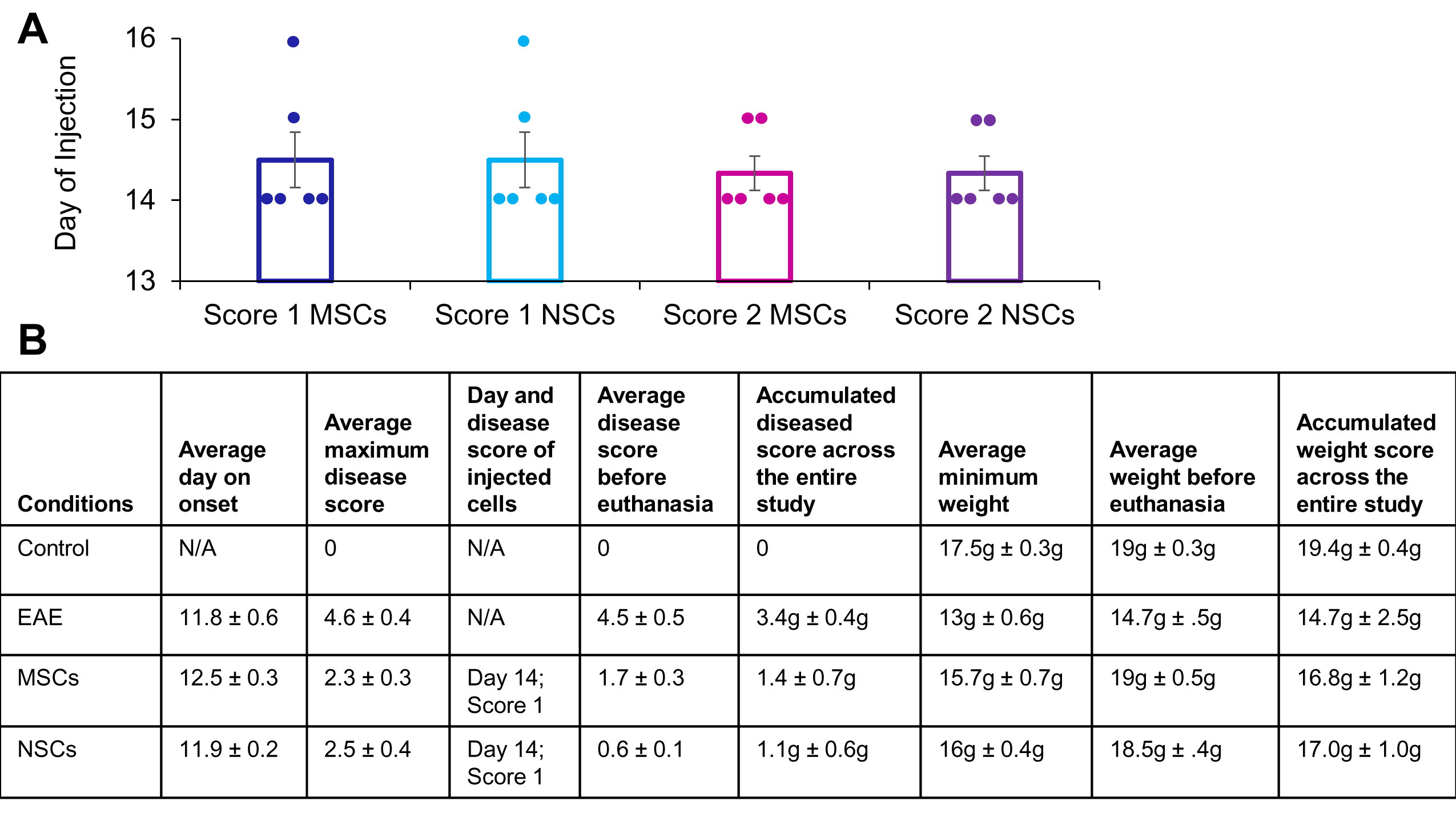

Supplement: Supplementary file 3 — Additional file 3. Detailed analysis of cell transplantation on EAE clinical symptoms and weight. (a) Graphical representation of the day the cells were transplanted for each EAE group. (b) Clinical parameters of the EAE mice prior to and after treatment with primitive MSCs or NSCs. [file 13287_2021_2563_MOESM3_ESM.tif]
